# The Effect of Including Sea Buckthorn Berry By-Products on White Chocolate Quality and Bioactive Characteristics under a Circular Economy Context

**DOI:** 10.3390/plants13192799

**Published:** 2024-10-05

**Authors:** Otilia Cristina Murariu, Florin Daniel Lipșa, Petru Marian Cârlescu, Gabriela Frunză, Marius Mihai Ciobanu, Irina Gabriela Cara, Florin Murariu, Florina Stoica, Aida Albu, Alessio Vincenzo Tallarita, Gianluca Caruso

**Affiliations:** 1 Department of Food Technology, ‘Ion Ionescu de la Brad’ Iasi University of Life Sciences, 700490 Iasi, Romania; otilia.murariu@iuls.ro (O.C.M.); petru.carlescu@iuls.ro (P.M.C.); marius.ciobanu@iuls.ro (M.M.C.); 2 Research Institute for Agriculture and Environment, ‘Ion Ionescu de la Brad’ University of Life Sciences, 700490 Iasi, Romania; irina.cara@iuls.ro (I.G.C.); florina.stoica@iuls.ro (F.S.); 3 Department of Agroeconomy, ‘Ion Ionescu de la Brad’ Iasi University of Life Sciences, 700490 Iasi, Romania; florin.murariu@iuls.ro; 4 Department of Control, Expertise and Services, ‘Ion Ionescu de la Brad’ Iasi University of Life Sciences, 700489 Iasi, Romania; aida.albu@iuls.ro; 5 Department of Agricultural Sciences, University of Naples Federico II, Via Università 100, Portici, 80055 Naples, Italy; alessiovincenzo.tallarita@unina.it (A.V.T.); gcaruso@unina.it (G.C.)

**Keywords:** *Hippophae rhamnoides* L., functional food, innovative products, rheological properties, colour, antioxidants, mineral composition

## Abstract

The by-products of the extraction of sea buckthorn (*Hippophae rhamnoides* L.) concentrated juice may represent a functional food ingredient for white chocolate production, as a rich source of bioactive compounds. The effects of six treatments derived from the factorial combination of two types of by-products (with oil or without oil) and three different concentrations (5%, 10%, and 15%), were assessed on rheological, quality, colour, antioxidant, and mineral properties of chocolate. The 15% addition of full powder led to the highest values of max firmness, total shear energy, shear energy, cohesiveness, gummosity, dry matter, and ABTS, compared to the untreated control, but the two highest concentrations of the oil-deprived powder resulted in the protein content increasing. The full powder addition always raised fat levels. Both the ‘L’ and ‘a’ colour component as well as total carotenoids, β-carotene, lycopene, and vitamin C increased with the rise of *H. rhamnoides* powder addition, compared to the untreated control. The opposite trend was shown by the ‘b’ colour component and pH, whereas polyphenols and antioxidant activity attained higher values with the oil-deprived powder. The content of potassium decreased upon the 15% addition of the *Hippophae rhamnoides* by-product powder, compared to the untreated control, whereas calcium and magnesium increased. The 15% *H. rhamnoides* full powder elicited the augmentation of phosphorus content in chocolate, compared to the untreated control, contrary to the effect of the oil-deprived powder on P and Zn. The employment of SBB by-products highlights the great potential for manufacturing innovative functional foods with high nutritional value, such as chocolate.

## 1. Introduction

The worldwide food market has had an increasing demand for functional food containing remarkable amounts of antioxidant components with highly beneficial impacts on human health [1]. Based on the definition of the EC Concerted Action on Functional Food Science in Europe (FUFOSE) [2], this is a food that beneficially affects one or more target functions in the body beyond adequate nutritional effects in a way that is relevant to either an improved state of health and well-being and/or reduction in risk of disease and is consumed as part of a normal food pattern (i.e., not a pill, a capsule, or any form of dietary supplement). In this respect, the integration of sea buckthorn fruits into innovative production chains showed advantageous effects, like in novel cheese [1], yoghurt, jam, and jelly as well as beverages [3]. Based on the existing literature, no studies regarding the addition of sea buckthorn to chocolate have been carried out so far; although, today’s food market needs New Product Development (NPD) projects, which are crucial to the success and progress of the related organization. White chocolate is defined as the product obtained from cocoa butter, milk, or milk products and sugars, which contains no less than 20% cocoa butter and no less than 14% dry milk solids obtained by partly or wholly dehydrating whole milk, semi- or full-skimmed milk, cream, or from partly or wholly dehydrated cream, butter, or milk fat, of which no less than 3.5% is milk fat [4]. However, a wide range of antioxidants, both phenolics and plant extracts, have been added in the chocolate manufacturing process [5,6,7,8,9,10,11,12]. Notably, in the latter respect, most of the research regarding the development of new matrices has been based on the replacement of sugar with various plant mixtures, such as stevia, as a key factor in the prevention of many diseases. Moreover, studies carried out in this field were based on the antimicrobial and antioxidant activity of some polyphenol extracts from different plants, such as carob (*Ceratonia silique* L.) in chocolate production [5] or sea buckthorn powder to obtain natural food at a low price with high nutritional properties. Innovations are also represented by gummy candies with the insertion of new substances either to gel or to replace sugars with natural flavourings and colourants, antioxidants, and other valuable ingredients. The creation of new food products has reshaped the confectionary industry, offering interesting new perspectives on sensorial, textural, and nutritional properties to fulfil consumer expectations [13].

Functional food products are also defined as “natural or processed foods containing effective and non-toxic amounts of bioactive compounds, which provide clinically proven and documented health benefits through the use of specific biomarkers to prevent human aging, treat chronic diseases or their symptoms” [14].

*Hippophae rhamnoides*, popularly called white sea buckthorn, is an over 2 billion-year-old shrub belonging to the Elaeagnaceae family, which is spread throughout Central Asia, Europe [15], and China either as a spontaneous plant or a crop, whose fruits are considered very health beneficial worldwide [16,17]. The overall market of *Hippophae rhamnoides* products is about 17 times bigger than that devoted to just berries, and this species has been widely adapting to different soil types, even the poorest and driest [18].

Sea buckthorn fruits (*Hippophae rhamnoides* L.) are rich in antioxidants and therapeutic compounds such as acid L-ascorbic, flavonoids, carotenes, volatile oils, vitamins, amino acids, and minerals [19]. In terms of antioxidant properties, the most important compounds are represented by flavonoids such as rutin, quercetin, kaempferol, myricetin, isorhamnetin [20], tocopherols, and carotenoids [21]. Furthermore, sea buckthorn displays remarkable antioxidant activities [22], and this is why in Latvia, sea buckthorn is referred to as Latvian Gold [17]. *Hippophae rhamnoides* berries are also an important bioactive food due to their nutritional and health-promoting properties like inflammation reduction, hair and skin regeneration, and anti-toxic effects [15]. Due to the high content of vitamin C and n-3, n-6 and n-9 polyunsaturateted fatty acids, sea buckthorn is used in many food industry products such as juice, alcohol drinks like wine and liquor, beer additives [23], jams, jellies, marmalades, sauces, oils, syrups, soft drinks [23], freeze-dried powders, milk tablets, teas, and preserved fruits [3]. The manufacturing of the mentioned products, as well as the entire agri-food supply chain, at a global level, generates high amounts of waste and by-products [15].

The present research proposes a new strategy to comprehensively address both the challenges of sustainable food systems related to the future world inhabitant rise and a possible agri-food resource deficiency, by valorising the sea buckthorn industry by-products. The latter goal is necessary for the sustainable use of resources preventing their negative impact on the environment, which is either the target of the European Green Deal [24] or the way to boost the profits of sea buckthorn manufacturers and growers [17].

Sea buckthorn berries ((SBB) *Hippophae rhamnoides* L.) were used as raw materials for juice extraction in Elexius manufacturing located in the Bacău region (Romania), and the by-products obtained were used in some innovative chocolate experimental products. Indeed, sea buckthorn powder is a versatile product with therapeutic and antioxidant properties that can be easily integrated into semi-viscous or solid food products and, therefore, can be employed for the development of new chocolate production chains with improved quality and nutritional characteristics.

This study aimed to valorize the fruit waste as dried powder derived from the sea buckthorn juice extraction in the Elexius unit to assess the most efficient technological practises and recipes to obtain novel chocolate with a high content of nutrients and bioactive compounds. Specifically, the effect of *Hippophae rhamnoides* by-products at different concentrations in their interactions with the presence or absence of oil was assessed on rheological, quality, colour, antioxidant, and mineral characteristics.

## 2. Results and Discussion

### 2.1. Rheological Parameters

As reported in Table 1, the increasing addition to white chocolate of *Hippophae rhamnoides* by-product powder, up to 15% concentration, resulted in rising values of the rheological characteristics analyzed, i.e., max firmness, total shear energy, shear energy, cohesiveness, and gummosity, compared to the untreated control. The best reaction to sea buckthorn application was recorded with the 15% addition of full powder, which did not significantly differ from the same percentage of oil-deprived powder in the cases of cohesiveness and gummosity. The untreated control always showed the lowest values, not significantly different from the 5% addition of full powder and also from the same percentage of oil-deprived powder in the cases of cohesiveness and gummosity.

Toker et al. [8] studied the effects on chocolate of the addition of omega 3 fatty acids (EPA/DHA) whose stability is affected by environment conditions such as temperature, light, and oxygen. Particularly, chocolate was enriched with encapsulated or microalgae-containing forms of eicosapentaenoic acid (EPA) and docosahexaenoic acid (DHA), after the conching process [8]. The chocolate samples had a shear-thinning behaviour, consistent with other research, which can be explained by the structural breakdown caused by the applied shear force [25] and alignment of the constituent molecules [26] present in the chocolates. Compared to the untreated control, the addition of different forms (microalgae/oil and powder) of EPA/DHA did not generally have a strong impact on chocolate rheological characteristics such as plastic viscosity, which represents an advantage for producers who can use the EPA/DHA source component without modifying the production process. The chocolate formulations produced with microencapsulated form led to the highest values of yield stress and plastic viscosity.

Chocolate rheological properties influence the efficiency of production chain, i.e., mixing, pumping, transportation, moulding, coating, sensory properties, and filling of rough surfaces [27], which relate to plastic viscosity [28]. Yield stress is affected by specific material surface, particle concentration and interactions, and emulsifiers [29]. Final hardness depends on plenty of factors such as recipe, manufacturing system, stability of fats, and, in this respect, sugar-free chocolate usually has a softer structure than ordinary chocolate [30]. In cocoa butter form V, fat triglycerides crystallize in triple chains showing a far greater thermodynamic stability than the double-chain system [29], as evidence of the relation between chocolate hardness and fatty acid profile [30]. Toker et al. [8] recorded a hardness range of 5.31 to 7.97 N, which was affected by the fatty acid composition, and the EPA/DHA source components led to decrease in hardness. Moreover, the higher level of polyunsaturated fatty acids (PUFAs) the lower melting temperature, but the addition of EPA/DHA had a negligible effect on chocolate melting properties.

Tolve et al. [9] inserted nanoparticles of vitamin D and magnesium calcium carbonate for the fortification of an innovative health beneficial chocolate produced with inulin and maltitol to replace sugars by a palm oil alternative diminishing saturated fatty acids concentration.

A significant viscosity increase in the No Palm No Sugar samples was elicited by Mg-CaCO_3_ nanoparticle addition. The Reference Brand (RB) chocolate, added with palm oil and sugar, had a higher viscosity than No Palm samples obtained with sunflower oil and shea butter. Neverthless, yield stress was higher in RB compared to other chocolate types. Notably, yield stress and plastic viscosity showed the same trends as the consistency index. The rheological behaviour of molten chocolate is affected by plenty of interrelated factors, i.e., humidity degree, ingredients such as fat, emulsifier and concentration, particle size distribution and shape, and processing procedure [31]. The lower yield stress and viscosity recorded in the basic formulation presumably relates to fat content (36% vs. 30–30.9%). Interparticle interactions, significantly influencing yield stress [32], are decreased upon the addition of the hydrophobic vitamin D into the formulate, potentially because of the higher availability of fat-like material coating all the particles [9]. The higher water content in samples produced using nanoparticles may also contribute to augment chocolate viscosity, though interparticle interactions and rearrangements are likely the major factors. Vitamin D + calcium added to No Palm No sugar chocolate lowered the viscosity compared to only Ca addition [9], suggesting more interparticle interactions encouraged by the lower availability of fats properly coating them [33].

### 2.2. Quality and Colour Parameters

The addition of 15% *Hippophae ramonoides* full powder to white chocolate led to a significantly dry matter increase compared to the untreated control and to the three treatments with oil-deprived powder, with the 15% addition of the latter application causing the lowest value (Table 2). The 10% and 15% additions of oil-deprived powder to chocolate resulted in the increase in protein content compared to the untreated control and to the other treatments regarding the highest sea buckthorn percentage. All the integrations with *Hippophae rhamnoides* by-product powders led to an increase in fats, compared to the untreated control, but each percentage of full powder was more effective than the corresponding percentage of the oil-deprived one (Table 2). The highest pH values were recorded with the untreated chocolate control and the lowest powder addition percentages. The ‘L’ colour component increased with the rise of *H. rhamnoides* powder addition compared to the untreated control (Table 2); the latter was not significantly different from the two lowest percentages of the full powder, which showed higher values than those associated with the oil-deprived one. The ‘a’ colour component increased with the rise of sea buckthorn powder application, while the ‘b’ component displayed the opposite trend; in both cases, the full powder led to higher values than those recorded with the oil-deprived one (Table 2).

The trends in the ‘a’ and ‘b’ colour components relate to the remarkable content of pigments contained in the *Hippophae* by-products added to the chocolate [15]. The ‘L’ and ‘b’ components were best affected by the lowest percentage of powder addition, whereas the ‘a’ component showed the opposite trend. Indeed, the powder added to chocolate derived from brownish fruits conferred a similar colour to the chocolate, which is associated with higher values of the component ‘a’ and, consequently, the components ‘L’ and ‘b’ were better affected by the lowest *Hippophae* powder percentage. No significant interactions between the two experimental factors arose on the three colour components examined.

The colour of food products is a critical factor for consumer acceptance and, in this respect, many parameters, i.e., brightness, shape, surface smoothness, translucency, and colour, can be used to characterize chocolate appearance [28]. Toker et al. [8] reported that the colour components measured showed narrow ranges of variation, similarly to previous findings [34]. The addition of EPA/DHA sources resulted in a darker chocolate than control [8] or redder [35]. However, the colour components can be affected by the origin and form of EPA/DHA sources added to chocolate [8], as well as by the chocolate composition and processing parameters during the production [36,37]. When the addition of the EPA/DHA sources did not have significant effect on the whiteness index, it depended on the fat bloom development, which damaged both visual and textural quality of chocolate [8].

### 2.3. Antioxidant Compounds and Activity

The 10% and 15% additions to white chocolate of full powder of sea buckthorn by-product and the 15% addition of oil-deprived powder resulted in a higher content of total carotenoids, β-carotene, and lycopene, compared to the untreated control (Table 3); moreover, the full powder was more effective than the oil-deprived one at the two highest percentages.

Similar trends to that described for the carotenoids were recorded for the polyphenols and antioxidant activity, but the oil-deprived powder generally led to higher values than the full powder (Table 3). The ABTS was increasingly enhanced by the rising powder addition percentages, compared to the untreated control, with the full powder producing better effects than the oil-deprived one. Vitamin C showed an increasing trend upon *H. rhamnoides* by-product powder addition, but the comparison between the two powder types was controversial (Table 3).

In this respect, the colour of *Hippophae* fruits and their by-products [15] reflects the high content of the mentioned pigments, which is transferred to chocolate during the processing.

Based on the mentioned results, the higher addition of *Hippophae* by-product powder percentage the higher effect of ODP on chocolate polyphenol and antioxidant activity, because the full powder has a greater polyphenol content than the oil-deprived powder [15].

Bolenz and Glöde [10] studied the addition of grape by-products, such as seeds and pomace, on chocolate. They found a positive correlation between grape pomace, total phenol content and antioxidative capacity, with samples containing 3.5% pomace showing about 236 mg GAE 100 g^−1^ d.w. The addition of either grape seeds or pomace containing polyphenols also increased their contents in final products in previous research [38,39]. Although chocolates naturally contain some polyphenols coming from cocoa particles, both ingredient types were able to significantly increase their amount together with antioxidative capacity. Grape seeds added less polyphenols but a more neutral taste, whereas the peels led to a product containing more polyphenols and showing darker colour together with an interesting fruity taste. As an alternative to pomace products, purifed extracts can be used, as described by Muhammad et al. [38] and Maier et al. [40]. Interestingly, Netzel et al. [41] showed that various phenolic compounds are metabolized, resulting in improved antioxidative capacities in the plasma and urea of tested people.

Tolve et al. [9] added vitamin D and magnesium-calcium carbonate nanoparticles to fortify chocolate and recorded the highest polyphenols concentration in Reference Brand chocolate; the addition of Vitamin D + Ca caused the lowest value because chocolate spread is characterized by a low percentage of cocoa and a high concentration in hazelnuts, which contain high polyphenols amount [42]. Loffredo et al. [43] demonstrated that commercial chocolate spread improves flow-mediated dilatation, and, thus, reducing the risk of cardiovascular diseases in smokers due to its antioxidants content. Based on the evaluation of the percentage of soluble compounds available for the absorption, that is the bioaccessibility [44], Tolve et al. [9] recorded a high polyphenol concentration in the Simulated Salivary Phase followed by a decrease in the Simulated Gastric Phase and a subsequent increase in the Simulated Intestinal Phase. The latter trend is similar to the pH one, with a dramatic reduction in the gastric tract and an increase in the neutral–basic intestine. Similar results have been reported by other researchers who worked on vegetable juices [45] and fruits [46]. Polyphenols are mainly found as esters, glycosides, and polymers in food, whose hydrolyzation by digestive enzymes before absorption is affected by several factors: mechanical destruction, food matrix, factors related to food processing, residence time in different gastrointestinal conditions, and enzymatic action as well as interactions with other constituents in the diet [47]. Tagliazucchi et al. [48] evaluated the bioaccessibility of grape polyphenols, reporting a release percentage of about 50% in Simulated Salivary Phase and 20% in Simulated Gastric Phase and Simulated Intestinal Phase. Hasni et al. [49] found that the interaction between milk caseins and polyphenols extracted from black tea can cause the formation of protein–polyphenol complexes based on non-covalent bonds, such as hydrogen or hydrophobic bonds, leading to a reduction in polyphenol bioaccessibility. The sharp increase in matrix degradation observed during the simulated gastric phase may be caused by acidity, while in the subsequent intestinal tract the further increase in matrix degradation is due to lipase activity. The fortification process did not influence the in vitro digestibility of the resulting products.

### 2.4. Mineral Elements

The content of potassium in white chocolate decreased upon the 15% addition of *Hippophae rhamnoides* by-product powder, either full or oil-deprived, compared to the untreated control, whereas calcium and magnesium increased, and sodium was not significantly affected by sea buckthorn powder application (Table 4). The 15% of *H. ramonoides* full powder elicited the augmentation of phosphorus content in chocolate, compared to the untreated control, whereas the oil-deprived addition caused a decrease in this mineral element. Zinc values decreased upon the application of 10 and 15% oil-deprived powder compared to the untreated control.

The different trends observed in the content of mineral elements under the experimental treatments applied can be explained by the laws of adsorption and cation exchange. In this respect, bivalent cations such as Ca^2+^ and Mg^2+^ are more strongly retained than the monovalent cation K^+^, because they have both a smaller ray and lower hydration degree with consequent higher electrostatic field.

Minerals are essential nutrients with different essential functions allowing important metabolic activities and maintenance of the human organism. In previous research [9], chocolate added with Mg Ca carbonate nanoparticles contained 9-fold more calcium and over twice magnesium content compared to other formulates. Besides the ingredient difference in the formulation, the mentioned variation may also relate to other factors, such as cultivation system of the vegetable’s ingredients [50]. In this respect, the hazelnut amount in chocolate can significantly influence the content of K, P, Ca, Mg, B, Cu and Mn [51]. Vitamin D addition to chocolate resulted in its retaining by over 90% leading to almost 15 times augmentation in the chocolate vitamin D content.

### 2.5. Sensorial Features

**Figure 1:**
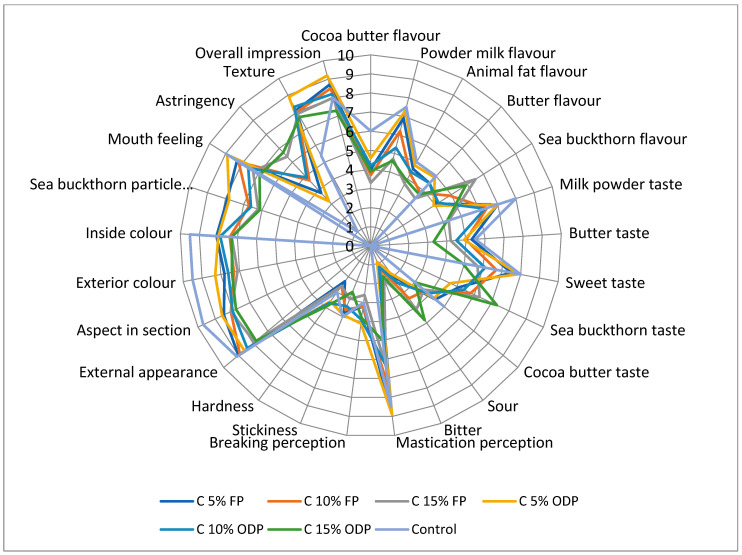
Sensorial features of chocolate added with *H. rhamnoides* by-products. C, chocolate; FP, *H. rhamnoides* by-product full powder; ODP, *H. rhamnoides* by-product oil-deprived powder.

As shown in Figure 1, compared to the untreated control, most of the sensorial features tested in white chocolate samples showed a decreasing trend with increasing addition from 5% to 15% of sea buckthorn by-product powder, both as FP and ODP: Cocoa butter flavour, powder milk flavour, animal fat flavour, butter flavour, milk powder taste, butter taste, sweet taste, cocoa butter taste, mastication perception, breaking perception, stickiness, external appearance, aspect in section, exterior colour, inside colour, thickness, Sea buckthorn particle finesse, mouth feeling, texture, and overall impression.

The values of the minority of sensorial features, i.e., sea buckthorn flavour and taste, sour, bitter, hardness, and astringency, increased with rising the addition percentage of *H. rhamnoides* by-product powder either containing or deprived from oil; the mentioned parameters attained the value 0 or 1 (astringency) in the control, except “hardness”. Notably, the values of butter taste were higher under the by-product full powder addition, whereas the breaking perception and hardness were higher when the by-product oil-deprived powder was added to chocolate. Rancid taste and thickness were not significantly affected by the experimental treatments.

## 3. Materials and Methods

### 3.1. Experimental Protocol and Raw Materials

Research was carried out at Iasi University ‘Ion Ionescu de la Brad’ in 2022 and 2023. The experimental protocol was based on the comparison between six treatments derived by the factorial combination of 2 types of sea buckthorn (*Hippophae rhamnoides* L.) by-product powders (with oil and without oil), and 3 different concentrations of addition to white chocolate (5%, 10%, and 15%), plus an untreated control, with three replicates. The effects of the experimental treatments were assessed on the rheological, quality, colorimetric, antioxidant, and mineral characteristics of chocolate.

Sea buckthorn berries (SBB (*Hippophae rhamnoides* L.)) that were organically grown, were used as raw materials for juice extraction in Elexius manufacturing from the Bacău region (Romania) and the by-products were used to produce innovative white chocolate products. The presence or absence of oil in *Hippophae rhamnoides* L. fruits relates to the quality characteristics of this species, which shows superior properties with high levels of antioxidant capacity, polyphenols, flavonoids, carotene, lycopene, vitamin C, total lipid and protein content, mineral components, and textural and colorimetric properties [15].

### 3.2. Sea Buckthorn Material, Fruit Processing and Chocolate Preparation

The fruits were harvested in mid-September, quickly frozen (within 27 min) at −40 °C, then stored until processing (about 3 months) at −25 °C.

The first step of processing was represented by juice production, after that the fruits were sorted, cleaned, and cold pressed to obtain juice with high antioxidant properties. The resulting by-products, represented by peels and seeds, were dried at a temperature of 45 °C, up to a humidity of 10%, for about 70 h.

The juice yield was 90%, and the remaining 10% was represented by sea buckthorn by-products, which was managed as follows: the residues were subjected to the drying process and grinding and sifting operations, allowing powder with sea buckthorn oil to be obtained; the residues underwent an oil extraction process to be reprocessed by drying, grinding, sieving, and, thus, resulting in powder sea buckthorn without oil.

White chocolate is a sugary product, which is susceptible to melting in the mouth without being able to detect the presence of solid particles, with a fine aroma and taste. These qualities, i.e., degree of dispersion, unctuousness and smell, are the result of physical and biochemical processes that take place during the processing of the main raw materials: powdered milk mass, sea buckthorn powder, cocoa butter, sugar, and flavourings. In order to achieve a sensation of non-detection of solid components, they must be smaller than 20–25 µm, which is the threshold for detection by the olfactory organs.

The experimental material was represented by six treatments named chocolate specialties, plus an untreated control, manufactured by moulding in the form of bars.

Based on international standards, chocolate is classified according to the sugar content into the following product categories: very sweet (57–60% sugar), sweet (45–57% sugar), semi-sweet (40–50% sugar), semi-bitter (maximum 45% sugar), bitter (maximum 42% sugar), and very bitter (maximum 30% sugar) [52].

Therefore, when establishing the recipe for the manufacturing of the sea buckthorn chocolate samples, the sugar content was considered, adding 30% in the present research, with fat content of the powdered milk being 26% and the proportion of fat from cocoa butter being 15%. The quality of the chocolate is correlated with the finesse of the particles of sea buckthorn powder, sugar, and possibly milk powder; its unctuousness depends on the fat content and the degree of dispersion of the solid particles and their wrapping in cocoa butter films. The taste is determined by the percentage of addition of sea buckthorn powder, sugar, milk powder, and flavouring; the smell is determined by the components involved in the recipe (cocoa butter, sea buckthorn powder, powdered milk, and flavourings).

### 3.3. Chocolate Processing

The raw materials, represented by water, caster sugar, milk powder, cocoa butter, sea buckthorn powder, and flavourings, were subjected to the conditioning process by weighing for a proper dosage, sifting to remove foreign bodies, and aeration. The cocoa butter was brought to 60–70 °C. Powdered milk was homogenized with sea buckthorn powder, simultaneously preparing the syrup from water and sugar by thermal treatment at 103.5 °C for 7–8 min. Next, the formation of the mixture occurred in the vat of a planetary mixer, where the optimal dispersion of the components and their homogenization took place, as well as the fluidization of the mass by the addition of cocoa butter, a process that is carried out by mixing them at a temperature between 65 and 70 °C.

The tempering stage of the chocolate mass was carried out to create conditions for the formation of crystallization centres of the stable form of cocoa butter by cooling the chocolate mass from 65 to 45 °C to 29 to 31 °C in about 40 min.

The white chocolate mass was subjected to the forming and modelling process by casting and filling in the form of bars, with the temperature of the forms of 30 °C, ensuring the vibration of the forms for 2–3 min to remove air bubbles from the cast chocolate mass. The removal of the excess chocolate mass and the cooling of the cast mass were applied to ensure the solidification of the mass and the formation of the chocolate structure. The cooling was carried out at a temperature of 2–4 °C for 20–30 min.

After 12 h of refrigerated storage, the chocolate was removed from the moulds, packed, and stored at 18–20 °C with 70–75% relative humidity for quality analysis.

### 3.4. Determination of Textural Properties

In order to determine the texture of white chocolate samples, a Mark 10^®^ (USA) texturometer was used. The texturometer cylindrical probe TA5 type, 12.7 mm in diameter and 35 mm height, was used for the measurements with a range of 100 and with a resolution of 0.05 N. A Warner Bratzler V-knife was used to cut the cobblestone samples. The moving velocity in the time of the insertion into the sample was 200 mm per minute, which is 3.33 mm per second. The MeasurePlus software model 15-1006 of the texturometer allowed us to record the graphs in force–deformation and force–time. The graphs were interpreted with the GraphPad Prism 9 software, and the results obtained helped to calculate the texture parameters of the analyzed chocolate samples.

To determine the texture, three bars with the following geometrical characteristics: 22 ± 1 mm average value for height, 87 ± 1 mm for length, and 37 ± 1 mm for width, were used from each chocolate sample, and each sample was tested in triplicate. Each sample was tested at a temperature of 20 ± 1 °C.

The mentioned rheological determinations were repeated three months after the production of *Hippophae rhamnoides* added chocolate, but the results have not been reported because they are not significantly different from those recorded at the first determination.

### 3.5. Determination of Total Dry Matter Content

The total dry matter content was determined by the oven drying method (103–105 °C) until constant weight in a forced air-drying oven (Biobase^®^, Jinan, China). The results were expressed as percentage of dry matter [53].

The dry matter determination as well as all the following quality analyses were repeated three months after the production of *Hippophae rhamnoides* added chocolate, but the results have not been reported because they are not significantly different from those recorded at the first determination.

### 3.6. Determination of Protein Content

Nitrogen content was determined by the Kjeldahl method using DK6 VELP Scientifica and UDK142 VELP Scientifica (VELP Scientifica, Usmate Velate MB, Italy). The amount of protein was calculated by multiplying the nitrogen percentage by the conversion factor of 6.25 [54,55].

### 3.7. Determination of Fat Content

The crude fat content was determined using the Soxhlet method. The extraction was carried out with Solvent Extractor SER 148 (VELP Scientifica, Usmate Velate MB, Italy) using hexane as solvent [54,55].

### 3.8. Determination of the Colour Components

The colour components of white chocolate samples were determined in replicate using a Konica Minolta Colorimeter-400 trichomatric reflectance colorimeter with Spectra Magic NX 1.3 software (Konica Minolta Sensing INC.^®^, Osaka, Japan), at an ambient temperature. The results were expressed according to the CIE Lab system components L*, a*, b* [56]. Measurements were made with an 8 mm optical glass aperture. Before the measurements, the equipment was calibrated using a standard white plate. The samples were evenly levelled and placed in a clear glass container for colorimetric analysis. The measurements were repeated three times at distinct points on the sample to ensure the accuracy of the results.

### 3.9. Extraction of Bioactive Substances from Chocolate Samples Added with Hippophae Rhamnoides *L*. By-Products

The phytochemicals from the white chocolate samples with the addition of sea buckthorn powder were extracted using the ultrasound-assisted extraction method. One g of sample was mixed with 10 mL of n-hexane/acetone solvent mixture (3:1, *v*/*v*, for ABTS method antioxidant activity, carotenoids) or 70% ethanol (only for total polyphenol extraction and antioxidant activity by the DPPH method) and was subjected to an ultrasound treatment for 30 min at a maximum of 32 °C and a frequency of 40 kHz. After recovering the resulting crude extract, it was centrifuged for 10 min at 5000 rpm at 4 °C. The supernatant was collected after separation and then analyzed to determine the amount of β-carotene, total carotenoids, and total polyphenols as well as the antioxidant activity (ABTS and DPPH).

The following antioxidant determinations were repeated three months after the production of *Hippophae rhamnoides* added chocolate, but the results have not been reported because they are not significantly different from those recorded at the first determination.

### 3.10. Determination of Carotenoid Compounds

The content in carotenoids (β-carotene and lycopene) was determined by the spectrophotometric method, measuring the absorbance at 450 nm, at 470 nm, and 503 nm [57] and calculated using the formula:

Carotenoids (mg/g) = (A × Mw × DF)/(m × l × ε) mg/g T.D.M

where, A is the absorbance of the sample; Mw is the molecular weight (536.88); DF is the dilution factor (if the sample was diluted); m is the weight/mass of the concentrated extract; l is the length of the optical path of the cuvette (1 cm for the quartz cuvette); and ε is the extinction coefficient, which is 2500 for the total carotenoids, 2590 for β-carotene, and 3450 for lycopene.

### 3.11. Determination of Total Polyphenol Content

The total polyphenol content was evaluated using the Folin–Ciocâlteu reagent method [58]. Particularly, 0.20 mL of the diluted sample (1:10) was added with 15.8 mL of distilled water and 1 mL of Folin–Ciocâlteu reagent. The mixture was shaken vigorously and allowed to react for 10 min, after which 3 mL of 20% sodium carbonate was added. The mixture was incubated for 60 min at room temperature. The absorbance was read by a spectrophotometer at the wavelength λ = 765 nm, using a UV-Vis spectrophotometer (Analytik Jena Specord 210 Plus, Jena, Germany). Gallic acid was used as a standard for the calibration curve, and the results obtained were expressed as dry matter (mg AG g^−1^).

### 3.12. Determination of Vitamin C Content

Vitamin C was quantitatively determined by a relatively simple method using 2,6-Dichlorophenolindophenol as described by Jones et al. [59]. Vitamin C was extracted from fresh samples with oxalic acid using a pinch of acid washed quartz sand. The supernatant was titrated against standard 2,6-Dichlorophenolindophenol, which had already been standardized with ascorbic acid [60]. The results were expressed as mg 100 g^−1^ of f.w.

### 3.13. Determination of Antioxidant Activity

The antioxidant activity was determined by the DPPH (2.2-diphenyl-1-picryhydrazyl) method. The preparation of the DPPH stock solution was carried out by dissolving 3.5 mg of DPPH in 100 mL of methanol. Then, 100 µL of the prepared sample extract was added with 3.9 mL of DPPH, with the blank consisting of the mixture of 100 µL methanol and 3.9 mL DPPH. It was left to stand for 30 min and then the readings were carried out at the absorbance of 515 nm. The determination of the antioxidant activity involved determining the inhibition for each sample to be analyzed using Equation (1), where the variations in the antioxidant capacity corresponding to the different samples were analyzed.

I(%) = (Ablank − Asample)/Ablank × 100(1)

where, Ablank is the absorbance of the control sample and Asample is the absorbance of the sample.

The results can also be expressed as µmol Trolox g^−1^ dry substance.

The determination of the antioxidant capacity using the radical cation ABTS^+^ is based on the ability of antioxidants to neutralize the ABTS^+^ radical, compared to a standard antioxidant, i.e., the vitamin E analogue Trolox [61]. ABTS^+^ is generated by oxidizing ABTS (2.2-azinobis 3-ethylbenzothiazoline-6- sulfonic acid) with 2.45 Mm potassium persulfate in the dark at 21 °C for 12–16 h. The ABTS stock solution was diluted with ethanol until the absorbance of 0.700 ± 0.020 at λ = 734 nm, using a UV-Vis spectrophotometer (Analytik Jena Specord 210 Plus, Jena, Germany). The results are expressed as inhibition (%ABTS) (2):

Inhibition (%ABTS) = (Ablank − Asample)/Ablank × 100(2)

where, A is the absorbance of the sample and Asample is the absorbance of the control sample (control sample).

The results can also be expressed as µmol Trolox/g dry matter substance.

### 3.14. Determination of Mineral Elements

For the evaluation of mineral components, the atomic absorption spectrometry (ContrAA 700, Analytik Jena, Jena, Germany) was used, with a flame atomizer system. The results were expressed in mg 100 g^−1^ d.w.

The measures of the mineral element contents (K, Ca, Mg, Na, P, Zn, and Fe) of the samples of white chocolates added with sea buckthorn were carried out using a MiniWAVE Microwave (SCP Science, Baie-d’Urfé, QC, Canada) digestion system equipped with a 50 mL Teflon vessel. A total of 1 g of the homogenized sample was weighed into a Teflon vessel and digested using a nitric (HNO_3_) and hydrogen peroxide H_2_O_2_ mixture (7:3). The digestion was performed under the following conditions: temperature, 190 °C; digestion time, 30 min; and microwave power, 1000 W. After cooling, the samples were carefully transferred into a 50 mL volumetric flask and diluted with ultrapure water until the mark. A blank sample was added in every digestion run, and each sample was prepared in triplicate.

### 3.15. Sensorial Features

For each white chocolate sample, by anonymous coding, a sensorial evaluation test was performed at the Department of Food Technology of “Ion Ionescu de la Brad” Iasi University (Romania), by a panel test of 15 specialists from the chocolate industry, composed of 30–50-year-old women and men sitting spatially apart to prevent opinion exchange. The temperature of the booths was set at 22 °C, while the preparation/serving room was set at 20 °C. Each of the mentioned specialists were provided with water, allowing for the removal of mouth residual material and taste, and they evaluated 10 g chocolate samples under neutral light (4000 K) over a required time. The experts’ opinions were reported in a specific form, including the following 27 sensorial variables, with a score scale from 0 (unpleasant) to 10 (pleasant): cocoa butter flavour, flavour powder milk, animal origin fats, butter flavour, sea buckthorn flavour, butter taste, sweet taste, sea buckthorn taste, cocoa butter taste, rancid taste, sour, bitter, tasteless, mastication perception, breaking perception, stickiness, hardness, external appearance, aspect in section, exterior colour, inside colour, thickness, sea buckthorn particle finesse, mouth feeling, astringency, texture, and overall impression.

Chocolate samples (two pieces per treatment, 1 cm × 1 cm of 7.3 g each) were served on a tray in a random order with 3-digit random codes for the evaluation. Water and crackers were served to panellists to cleanse the palate between two consecutive samples. To determine the texture, three bars (22 ± 1 mm thick, 87 ± 1 mm long, and 37 ± 1 mm wide) were used from each chocolate treatment and tested in triplicate at the temperature of 20 ± 1 °C.

### 3.16. Statistical Analysis

Data were processed by analysis of variance (ANOVA) and mean separations were performed through Duncan’s test, with reference to 0.05 probability level, using SPSS software version 29.

## 4. Conclusions

This research aimed to assess and valorise the *Hippophae rhamnoides* by-products resulting from the extraction of concentrated juice, which are rich in bioactive substances, and their addition to white chocolate to produce a functional food. From the comparison between six treatments derived by the factorial combination of two different types of the mentioned by-products (oil containing full powder or oil-deprived powder) and three different concentrations (5%, 10%, and 15%), it arose that the highest addition of full powder led to the enhancement of the rheological properties and dry matter. However, all the concentrations of sea buckthorn by-product full powder resulted in the increase in fats; however, the two highest concentrations of the oil-deprived powder elicited an increase in protein. The highest addition of both the types of *H. rhamnoides* powder enhanced the brightness (L*) as well as the tendency to red (a*) and blue (b*) colour of white chocolate. The highest percentage of sea buckthorn by-product powder favoured the accumulation of most of the analyzed antioxidants and ABTS in chocolate, though the oil-deprived powder showed a better effect on polyphenols and antioxidant activity. The effect of the *Hippophae rhamnoides* by-product addition to white chocolate mineral content was controversial, as potassium level decreased with the powder rise, whereas calcium, magnesium, and phosphorus increased.

In most cases, the values of sensorial features recorded in the untreated white chocolate samples were higher than those corresponding to the sea buckthorn by-product powder addition, and decreased with increasing the *H. rhamnoides* waste percentage, both as full and oil-deprived powder; the minority of sensorial parameters showed the opposite trend.

From the present research, it can be inferred that the valorisation of sea buckthorn by-products derived from concentrated juice extraction represents an interesting strategy to produce innovative functional foods with highly beneficial impacts on human health, such as chocolate.

## Figures and Tables

**Table 1:** Rheological characteristics of chocolate added with *H. rhamnoides* by-products.

Type of *Hippophae rhamnoides* Powder (TP) × Percentage of Addition (PA)	Texture (N)	A1—Total Shear Energy (mJ)	A2—Shear Energy (mJ)	Cohesiveness	Gummosity (N)
Chocolate with no addition	7.0 ± 0.3 e	145 ± 9 e	72 ± 2 e	0.50 ± 0.01 c	0.49 ± 0.02 d
Chocolate with 5% full powder (FP) addition	7.1 ± 0.2 e	151 ± 11 de	76 ± 5 e	0.50 ± 0.01 c	0.50 ± 0.02 cd
Chocolate with 10% full powder (FP) addition	7.7 ± 0.4 e	166 ± 13 d	92 ± 1 d	0.55 ± 0.02 ab	0.54 ± 0.02 ac
Chocolate with 15% full powder (FP) addition	13.9 ± 0.5 a	290 ± 18 a	169 ± 8 a	0.58 ± 0.02 a	0.58 ± 0.02 a
Chocolate with 5% oil-deprived powder (ODP) addition	8.7 ± 0.3 d	200 ± 12 c	109 ± 5 c	0.51 ± 0.02 bc	0.48 ± 0.02 d
Chocolate with 10% oil-deprived powder (ODP) addition	10.3 ± 0.4 c	216 ± 13 c	111 ± 8 c	0.52 ± 0.02 bc	0.52 ± 0.02 bd
Chocolate with 15% oil-deprived powder (ODP) addition	11.9 ± 0.4 b	244 ± 15 b	131 ± 6 b	0.55 ± 0.02 ab	0.55 ± 0.02 ab

Within each column, mean values followed by different letters are significantly different at *p* ≤ 0.05 according to Duncan’s test.

**Table 2:** Quality and colour characteristics of chocolate added with *H. ramonoides* by-products.

Type of *Hippophae rhamnoides* Powder (TP) × Percentage of Addition (PA)	Dry Matter (%)	Proteins (%)	Fats (%)	pH	L	A	B
Chocolate with no addition	93 ± 6 b	11.3 ± 0.4 c	18 ± 1 e	6.2 ± 0.1 a	44 ± 1 e	5.7 ± 0.1 e	36 ± 1 a
Chocolate with 5% full powder (FP) addition	96 ± 5 ab	11.5 ± 0.4 bc	21 ± 1 c	6.2 ± 0.2 a	45 ± 0 e	7.6 ± 0.1 c	34 ± 2 a
Chocolate with 10% full powder (FP) addition	98 ± 7 ab	11.5 ± 0.5 bc	24 ± 1 b	6.1 ± 0.3 a	45 ± 1 de	7.6 ± 0.2 c	25 ± 2 c
Chocolate with 15% full powder (FP) addition	99 ± 7 a	11.8 ± 0.6 bc	28 ± 1 a	5.1 ± 0.2 bc	57 ± 1 a	9.8 ± 0.1 a	19 ± 1 e
Chocolate with 5% oil-deprived powder (ODP) addition	93 ± 3 b	11.5 ± 0.4 bc	19 ± 1 de	6.0 ± 0.2 a	50 ± 1 cd	6.0 ± 0.1 e	29 ± 1 b
Chocolate with 10% oil-deprived powder (ODP) addition	91 ± 2 bc	12.3 ± 0.4 ab	20 ± 1 cd	5.6 ± 0.2 b	50 ± 1 bc	6.9 ± 0.1 d	21 ± 1 d
Chocolate with 15% oil-deprived powder (ODP) addition	86 ± 1 c	12.7 ± 0.4 a	25 ± 1 b	4.8 ± 0.2 c	54 ± 1 ab	9.1 ± 0.1 b	19 ± 1 e

Within each column, mean values followed by different letters are significantly different at *p* ≤ 0.05 according to Duncan’s test.

**Table 3:** Antioxidant characteristics of chocolate added with *H. rhamnoides* by-products.

Type of *Hippophae rhamnoides* Powder (TP) × Percentage of Addition (PA)	Total Carotenoids (mg g^−1^ f.w.)	β-Carotene (mg g^−1^ f.w.)	Lycopene (mg g^−1^ f.w.)	Total Polyphenols (mg g^−1^ f.w.)	Antioxidant Activity (% Inhibition)	ABTS	Vitamin C (mg 100 g^−1^ f.w.)
Chocolate with no addition	1.1 ± 0.1 d	0.6 ± 0.0 d	0.3 ± 0.0 d	0.3 ± 0.1 d	8 ± 1 d	39 ± 2 f	81 ± 5 e
Chocolate with 5% full powder (FP) addition	1.3 ± 0.1 d	0.9 ± 0.1 d	0.3 ± 0.1 d	0.3 ± 0.0 d	8 ± 1 cd	46 ± 2 d	88 ± 7 e
Chocolate with 10% full powder (FP) addition	3.3 ± 0.2 b	2.6 ± 0.3 b	1.2 ± 0.2 b	0.3 ± 0.1 d	10 ± 1 c	59 ± 3 c	154 ± 9 c
Chocolate with 15% full powder (FP) addition	6.3 ± 0.4 a	4.8 ± 0.4 a	2.2 ± 0.3 a	0.6 ± 0.1 b	18 ± 2 a	70 ± 4 a	264 ± 13 b
Chocolate with 5% oil-deprived powder (ODP) addition	1.2 ± 0.1 d	0.7 ± 0.1 d	0.3 ± 0.0 d	0.4 ± 0.0 c	9 ± 1 cd	41 ± 3 ef	88 ± 8 e
Chocolate with 10% oil-deprived powder (ODP) addition	1.3 ± 0.1 d	0.8 ± 0.1 d	0.4 ± 0.0 d	0.7 ± 0.1 a	14 ± 1 b	45 ± 2 de	132 ± 11 d
Chocolate with 15% oil-deprived powder (ODP) addition	2.2 ± 0.1 c	1.5 ± 0.2 c	0.7 ± 0.1 c	0.7 ± 0.1 a	20 ± 1 a	65 ± 2 b	286 ± 10 a

Within each column, mean values followed by different letters are significantly different at *p* ≤ 0.05 according to Duncan’s test.

**Table 4:** Mineral characteristics of chocolate added with *H. rhamnoides* by-products.

Type of *Hippophae rhamnoides* Powder (TP) × Percentage of Addition (PA)	K (mg 100 g^−1^)	Ca (mg 100 g^−1^)	Mg (mg 100 g^−1^)	Na (mg 100 g^−1^)	P (mg 100 g^−1^)	Zn (mg 100 g^−1^)
Chocolate with no addition	69 ± 4 a	144 ± 6 b	63 ± 2 d	109 ± 16	38 ± 2 bc	1.70 ± 0.08 a
Chocolate with 5% full powder (FP) addition	68 ± 3 a	151 ± 7 ab	64 ± 3 d	111 ± 16	38 ± 2 bc	1.64 ± 0.07 ab
Chocolate with 10% full powder (FP) addition	68 ± 3 ab	155 ± 7 ab	66 ± 3 cd	107 ± 16	41 ± 2 b	1.60 ± 0.04 ac
Chocolate with 15% full powder (FP) addition	63 ± 3 b	158 ± 6 a	74 ± 2 b	109 ± 16	58 ± 2 a	1.58 ± 0.04 ac
Chocolate with 5% oil-deprived powder (ODP) addition	66 ± 4 ab	145 ± 7 b	65 ± 3 cd	110 ± 17	36 ± 2 c	1.68 ± 0.08 ab
Chocolate with 10% oil-deprived powder (ODP) addition	63 ± 4 b	159 ± 7 a	70 ± 3 bc	107 ± 17	27 ± 2 d	1.54 ± 0.09 b
Chocolate with 15% oil-deprived powder (ODP) addition	61 ± 3 b	160 ± 7 a	82 ± 3 a	109 ± 17	17 ± 2 e	1.47 ± 0.09 c
				n.s.		

n.s, not significant; within each column, mean values followed by different letters are significantly different at *p* ≤ 0.05 according to Duncan’s test.

## Data Availability

All the data are available upon request.
